# Hedgehog Signaling Antagonist Promotes Regression of Both Liver Fibrosis and Hepatocellular Carcinoma in a Murine Model of Primary Liver Cancer

**DOI:** 10.1371/journal.pone.0023943

**Published:** 2011-09-02

**Authors:** George M. Philips, Isaac S. Chan, Marzena Swiderska, Vanessa T. Schroder, Cynthia Guy, Gamze F. Karaca, Cynthia Moylan, Talaignair Venkatraman, Sebastian Feuerlein, Wing-Kin Syn, Youngmi Jung, Rafal P. Witek, Steve Choi, Gregory A. Michelotti, Fatima Rangwala, Elmar Merkle, Christopher Lascola, Anna Mae Diehl

**Affiliations:** 1 Division of Gastroenterology, Department of Medicine, Duke University Medical Center, Durham, North Carolina, United States of America; 2 Department of Genetics, The University of North Carolina School of Medicine, Chapel Hill, North Carolina, United States of America; 3 Department of Surgery, Duke University Medical Center, Durham, North Carolina, United States of America; 4 Department of Pathology, Duke University Medical Center, Durham, North Carolina, United States of America; 5 Department of Radiology, Duke University, Durham, North Carolina, United States of America; 6 Department of Biological Science, Pusan National University, Pusan, Korea; 7 Divisions of Cell Therapy, Hematology and Medical Oncology, Duke University Medical Center, Durham, North Carolina, United States of America; University of Medicine and Dentistry of New Jersey, United States of America

## Abstract

**Objective:**

Chronic fibrosing liver injury is a major risk factor for hepatocarcinogenesis in humans. Mice with targeted deletion of Mdr2 (the murine ortholog of MDR3) develop chronic fibrosing liver injury. Hepatocellular carcinoma (HCC) emerges spontaneously in such mice by 50–60 weeks of age, providing a model of fibrosis-associated hepatocarcinogenesis. We used Mdr2^−/−^ mice to investigate the hypothesis that activation of the hedgehog (Hh) signaling pathway promotes development of both liver fibrosis and HCC.

**Methods:**

Hepatic injury and fibrosis, Hh pathway activation, and liver progenitor populations were compared in Mdr2^−/−^ mice and age-matched wild type controls. A dose finding experiment with the Hh signaling antagonist GDC-0449 was performed to optimize Hh pathway inhibition. Mice were then treated with GDC-0449 or vehicle for 9 days, and effects on liver fibrosis and tumor burden were assessed by immunohistochemistry, qRT-PCR, Western blot, and magnetic resonance imaging.

**Results:**

Unlike controls, Mdr2^−/−^ mice consistently expressed Hh ligands and progressively accumulated Hh-responsive liver myofibroblasts and progenitors with age. Treatment of aged Mdr2-deficient mice with GDC-0449 significantly inhibited hepatic Hh activity, decreased liver myofibroblasts and progenitors, reduced liver fibrosis, promoted regression of intra-hepatic HCCs, and decreased the number of metastatic HCC without increasing mortality.

**Conclusions:**

Hh pathway activation promotes liver fibrosis and hepatocarcinogenesis, and inhibiting Hh signaling safely reverses both processes even when fibrosis and HCC are advanced.

## Introduction

Hepatocellular carcinoma (HCC) is an insidious cancer that accounts for up to 1 million deaths a year and is the third leading cause of cancer deaths worldwide [1]. Cirrhosis, a consequence of progressive liver injury and fibrosis, is the single largest risk factor for HCC [2]. An altered wound healing response to chronic liver injury, with resultant dysregulated activation of myofibroblasts and progenitor cell populations, has been implicated in cirrhosis pathogenesis and eventual carcinogenesis [3].

Loss-of-function mutations in the hepatocyte canalicular phospholipid flippase MDR3 (ABCB4) have been associated with a wide range of human biliary diseases including progressive familial intrahepatic cholestasis type 3 (PFIC3), cholestasis of pregnancy, drug induced cholestasis and an adult biliary cirrhosis with features similar to primary sclerosing cholangitis (PSC) [4], [5], [6], [7]. Mice with a targeted deletion of Mdr2 (the murine ortholog of MDR3) lack the liver-specific P-glycoprotein that transports phosphatidylcholine (PC) across the canalicular membrane. The absence of phospholipids in bile results in progressive sclerosing cholangitis with accompanying portal inflammation, ductular proliferation and portal fibrosis. Liver injury manifests shortly after birth and hepatocellular carcinomas emerge spontaneously between 50 to 60 weeks of age [8], [9], [10], [11], [12], [13], [14]. Unlike xenograft models that are widely utilized to examine mechanisms of- and treatments for- HCC, Mdr2^−/−^ mice provide a model that parallels the natural evolution of HCC on a background of chronic inflammation, liver injury and fibrosis [15].

Gene expression analyses in Mdr2-deficient mice and heterozygote controls have demonstrated robust and sustained induction of multiple adaptive mechanisms that control cellular responses related to oxidative stress, inflammation, lipid metabolism, and proliferation, prompting speculation that these processes contribute to hepatocarcinogenesis [16]. Although not formally assessed by earlier studies of Mdr2-deficient mice, another pathway that might play a role in fibrosis-associated hepatocarcinogenesis is Hedgehog (Hh), because Hh signaling has been implicated in both fibrogenic repair of liver injury and HCC.

The Hh pathway is an evolutionarily conserved signaling pathway that is activated when Hh ligands (Sonic hedgehog and Indian hedgehog) bind to Patched (Ptc), a transmembrane receptor that is expressed on the surface of Hh-responsive cells. Upon ligand binding, Ptc is inactivated, relieving its repression of Smoothened (Smo), a trans-membrane protein that mediates Hh signaling inside the cell. Smo activation culminates in the nuclear localization of Gli-family transcription factors, Gli1, Gli2, and Gli3, which, in turn, regulate downstream gene expression. The pathway is quiescent in normal liver [17], [18], but becomes reactivated as a repair mechanism in chronic liver injury [19], [20], [21], [22], [23]. Hh ligands promote the growth and viability of myofibroblasts, the accumulation of which leads to abnormal liver repair, fibrosis and eventual cirrhosis [24], [25]. Hh ligands also serve as viability and proliferative factors for liver epithelial progenitors, and expansion of this compartment has been linked to the formation and maintenance of hepatocellular carcinomas [26]. The possibility that Hh pathway activation contributes to hepatocarcinogenesis is supported by the fact that Sonic hedgehog (Shh) ligand expression is noted in approximately 60% of human HCCs, and expression of the Hh-regulated genes, Gli1 and Ptc, occurs in 50% of human tumors [27], [28], [29], [30].

Based on these observations, we postulated that Hh pathway activation contributes to the pathogenesis of both liver fibrosis and fibrosis-associated HCC. To test this hypothesis, we treated aged Mdr2-deficient mice with the Hh pathway inhibitor, GDC-0449, a small-molecule inhibitor that binds to Smoothened (SMO). This agent was selected because of its human safety profile in phase 1 trials, as well as its effectiveness in solid organ tumors like basal cell carcinoma (BCC) and medulloblastoma [31], [32], [33]. Our aims were to determine whether or not mice with advanced liver disease and HCCs would tolerate Hh pathway inhibition and experience improvements in liver fibrosis and/or tumor burden.

## Methods

### Mice

Mdr2^−/−^ mice were a gift from Dr. Detlef Schuppan (Beth Israel Deaconess Medical Center, Boston, MA). Age matched FVB/NJ wild type mice were obtained from Jackson Laboratory (Bar Harbor, ME). All mice were housed with a 12-h light-dark cycle and given water and standard chow *ad libitum*. At ages, 2, 4, 12, 36 52 and 62-weeks, mice were sacrificed under general anesthesia. Liver weight and body weight were recorded, serum and liver tissue were collected. Animal studies were approved by the Institutional Animal Care and Use Committee as governed by the National Institute of Health's “Guide for the Care and Use of Laboratory Animals”, Duke University Animal Welfare Assurance Number A3195-01.

### Serum AST/ALT determination

Serum aspartate aminotransferase (AST) and alanine aminotransferase (ALT) were measured using kits commercially available from Biotron Diagnostics (66-D and 68-D respectively; Hemet, CA) according to the manufacturers' instructions.

### Western Blot

Total protein was extracted from snap frozen whole liver tissue in RIPA buffer. Samples were pooled by age (2–4 samples per age group) except as noted. Western blot analysis was performed as previously described. Membranes were probed with the following primary antibodies: Sonic hedgehog (sc-9024; Santa Cruz Biotechnology, Santa Cruz, CA), Indian hedgehog (ab39634; Abcam), β-actin (sc-47778, Santa Cruz Biotechnology), Gli2 (18-732-292462, Genway, San Diego, CA). All antibodies were diluted 1∶1000 and were incubated at 4°C overnight. Western blot images were acquired using a FluorChem HD2 digital darkroom system (Cell Biosciences, Santa Clara, California).

### Immunohistochemistry

4 µm formalin-fixed, paraffin-embedded samples were dewaxed and rehydrated. To evaluate tissue architecture, slides were stained with hematoxylin and eosin (H&E) and Sirius red per standard protocol. For immunohistochemistry, slides were incubated in 3% hydrogen peroxide/methanol. Antigen retrieval was performed by heating in 10 mM sodium citrate buffer or 0.25% pepsin (K19; Invitrogen, Carlsbad, CA) for 10 minutes. Sections were blocked (Dako Envision, Carpinteria, CA) and incubated with primary antibodies overnight at 4°C: glioblastoma-2 (Gli-2; 18-732-292462; 1∶2000; Genway, San Diego, CA); cytokeratin 19 (Troma-III; Hybridoma Bank, Iowa City, IA; 1∶500); α-fetoprotein (AFP) (Dako, 1∶1000); Indian hedgehog (Abcam; 1∶750, Cambridge, MA); Polymer-HRP anti-rabbit (K4003; Dako) or anti-mouse (K40011; Dako) or MACH3 mouse AP polymer kit (MP530, Biocare Medical, Concord, California, USA) were used as secondary antibodies. 3,3′-Diaminobenzidine (DAB) Substrate Chromogen System (K3466; Dako) and/or Ferangi Blue (Biocare) was employed in the detection procedure. Omitting primary antibodies from the reactions eliminated staining which demonstrated staining specificity. Images were acquired on an Olympus IX71 (Tokyo, Japan) inverted microscope using the DP2-BSW (Olympus) image acquisition software system.

### Quantitative Real-Time Reverse Transcription PCR

RNA was isolated from whole liver, as well as from resected tumor specimens had standard TriZol extraction as has been previously described [24]. A table of primers has been included in the Supplementary Materials.

### Morphometry

Formalin-fixed liver and tumor sections were stained for CD44 and osteopontin as described above, and were quantified by morphometric analysis with MetaView software (Universal Imaging, Downington, PA). A minimum of 5 randomly selected 10× or 20× fields/section were evaluated for each mouse.

### Quantitative Immunohistochemical Analysis

Formalin-fixed liver and tumor sections were costained for Gli2 and CK19. A minimum of 5 ductular regions, 20× fields per section, were evaluated for each mouse by counting the number of cells co-labelled.

### Hepatic hydroxyproline assay

The hydroxyproline content in whole liver specimens was quantified colorimetrically as previously reported [34].

### GDC-0449 treatment

In the initial, dose-finding cohort of animals, 16 Mdr2^−/−^ mice (age 57–68 weeks) were assigned to treatment with vehicle control (DMSO, n = 5), 20 mg/kg GDC-0449 (n = 5), or 40 mg/kg GDC-0449 (n = 6). Male-female ratios were balanced between the groups. GDC-0449 (Selleck Chemicals, Houston, TX) was freshly constituted daily in DMSO. All mice were given a daily intraperitoneal injection for 9 days. On day 10, animals were sacrificed. Samples were collected as above. A second cohort of 20 Mdr2^−/−^ mice (age 51–59 weeks of age) were subjected to whole body magnetic resonance imaging to assess tumor burden and then pairs of mice with comparable tumor burdens were assigned to treatment with either DMSO (control, n = 10) or 40 mg/kg GDC-0449 (n = 10). Drugs were delivered as described above; MRI scanning was repeated after the 9^th^ day of treatment; mice were sacrificed for necropsy and tissue harvest.

### Pathology

H&E and sirius red staining of liver sections was evaluated by a board-certified pathologist. Representative sections of tumor and non-tumor tissue were examined. Tumor tissue was defined as grossly visible nodules that were at least 10 mm in size and resectable.

### Abdominal magnetic resonance imaging

Animals were assigned a blinding code, which was maintained during magnetic resonance (MR) data acquisition and analysis. MR mouse liver imaging was performed on a 7T Bruker Biospec 70/30 horizontal bore system (Billerica, MA). Animals were lightly anesthetized under isofluorane with continuous monitoring and maintenance of physiological parameters throughout the imaging session (∼60 min for each animal). Axial and coronal 2D T2-weighted fast spin echo images (TURBO-RARE, TE/TR = 11/4200 ms with 1 mm slice thick, matrix = 256×256 and FOV of 2.4 cm×2.4 cm, 5 averages, 0.0 mm interslice gap) images were first obtained for screening purposes and supplemental anatomic information. For directed tumor volumetric analysis, 64 contiguous 500 µM thick 3D FSE proton density images biased towards T1 weighting (TURBO-RARE, TE/TR = 9/1500 ms, matrix = 256×256×64 and FOV 2.2 cm×2.2 cm×2.2 cm, 25 minutes duration) were acquired. All imaging sequences were performed using respiratory gating.

Volumetric analysis from MR data sets was performed in Osirix software, an open source image processing application developed and maintained by Pixmeo (Geneva, SUI). Liver tumors were manually segmented in each animal by a board-certified radiologist (blinded to treatment) post-treatment. Selected areas were reviewed for consistency on coronal and sagittal representations, and cross-correlated with axial 2D FSE images. For volumetry, two separate volume measurements were obtained for each lesion, with the average volume then taken. Interrater reliability (kappa value) was = 0.97. T1 and T2-weighted scanning sequences were performed.

### Statistical analyses

Results expressed as mean±SD. Significance established using the Student's t-test. Differences were considered significant when p<0.05.

## Results

### Progressive accumulation of Hh-responsive cells in Mdr2^−/−^ mice

**Figure 1:**
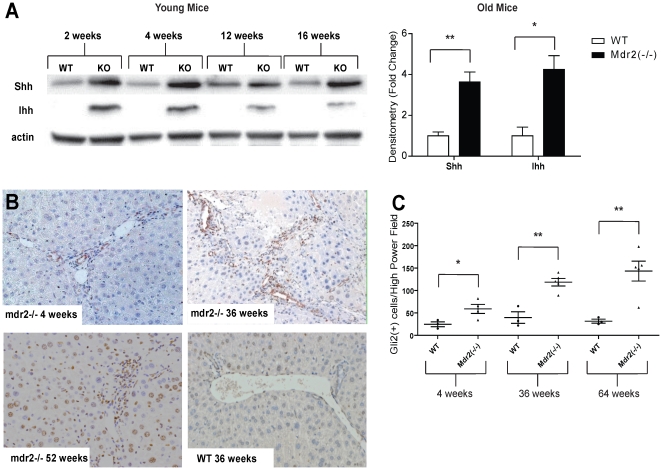
Increased hepatic Hedgehog (Hh) pathway activity in Mdr2^−/−^ mice. **A.** Western blot analysis for Sonic Hedgehog (SHh) and Indian Hedgehog (IHh) in whole liver extracts pooled from young Mdr2^−/−^ mice (2–16 weeks old), old (36–64 weeks old) Mdr2^−/−^ mice and age-matched controls (n = 3–5 mice/group/time point). A representative Western blot demonstrating results from young mice is shown. Mean±SEM data from older mice are graphed. **B.** Representative liver sections stained to demonstrate Gli2 from young (4 wk old), middle aged (36 wk old) and old (52 wk old) Mdr2^−/−^ mice. Little Gli2 expression was noted in wildtype mice at any age, so results from a representative 36 wk old wild type mouse is shown. (10×) **C.** Quantitative Gli2 immunohistochemical data. The number of nuclear Gli2(+) cells was counted in at least 5 high power fields (HPF) per liver section in Mdr2^−/−^ mice and wild type controls at 4, 36 and 64 weeks of age (n = 3–4 mice/group/time point) and mean±SEM are graphed. *p<0.05, **p<0.01 in Mdr2^−/−^ groups vs. respective controls.

Various forms of acute and chronic liver injury induce hepatic expression of Hh ligands and activation of Hh responsive cells. Serum levels of AST and ALT were consistently higher in Mdr2^−/−^ mice than age-matched controls (**Figure S1**), confirming that the knockout mice had chronic liver injury. Thus, we surveyed livers from Mdr2^−/−^ mice for Hh pathway activation. Compared to liver protein lysates from wild type controls, lysates from Mdr2^−/−^ mice demonstrated an increase in Sonic hedgehog (Shh) and Indian hedgehog (Ihh) by Western blot analysis. This was apparent at the first time point examined (2 weeks after birth) and maintained throughout the lifespan of the animals (up to 64 weeks of age) (**Figure 1A**). Immunohistochemistry for the Hh-regulated transcription factor, Gli2, demonstrated progressive, age-related accumulation of cells with nuclear Gli2 staining in Mdr2^−/−^ mice. Such Gli2-positive cells included stromal cells, as well as hepatocytic and ductular cells (**Figure 1B–C**). Together, these data suggest that the Hh pathway is activated in chronically injured livers of Mdr2-deficient mice, resulting in progressive expansion of various Hh-responsive cell populations.

### Treatment of Mdr2^−/−^ mice with the Smoothened inhibitor, GDC-0449, is safe and decreases Hedgehog pathway signaling

To determine the appropriate dose of GDC-0449, a pilot study was done with 16 aged (57–68 week old) Mdr2^−/−^ mice. Animals were given a daily intraperitoneal injection of 20 mg/kg GDC-0449 (n = 5), 40 mg/kg GDC-0449 (n = 6), or DMSO vehicle control (n = 5) for 9 days and then sacrificed. There was no statistical difference in mortality between the control and high dose (40 mg/kg) GDC-0449 groups. At sacrifice, liver/body weight ratios of mice in the three groups were also similar, suggesting that a relatively short course of systemic treatment with GDC-0449 at 40 mg/kg is well tolerated by mice with advanced liver disease and HCC (**Figures S2A–B**). Western blot analysis of liver lysates from this cohort of animals demonstrated that GDC-0449 treatment caused a decrease in Shh ligand levels at the higher dose, and Gli2 expression attenuation at both doses (**Figure S2C**).

### Hedgehog signaling inhibition with GDC-0449 abrogates effects of Hh signaling on target gene expression

**Figure 2:**
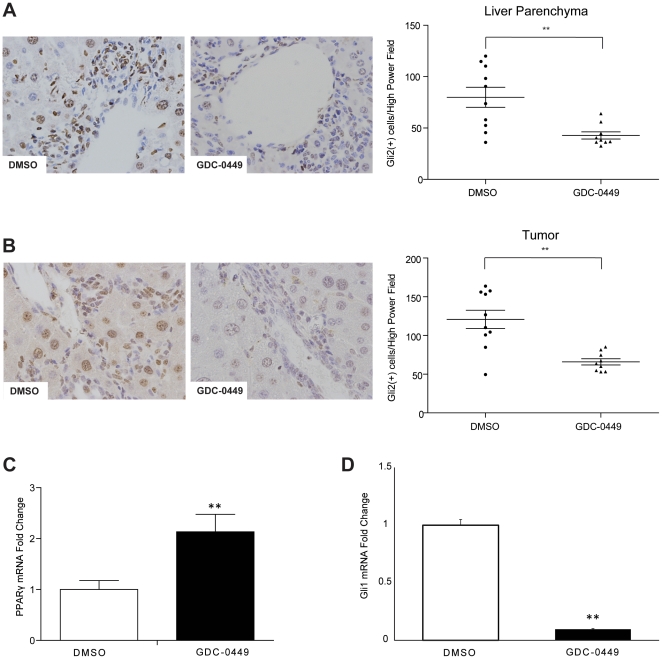
Hedgehog (Hh) inhibitor, GDC-0449, abrogates effects of Hh signaling within liver parenchyma and HCC nodules. **A.** Liver sections stained for Gli2 from representative DMSO- and GDC-0449- treated mice (40×). Quantitative Gli2 immunohistochemistry data in non-tumor livers of mice treated with DMSO or GDC-0449 (n = 9–10/group) are graphed as mean±SEM (**p<0.01. The number of ductular cells with Gli2 positive staining were counted in each portal tract/section under 40× magnification. **B.** Tumor sections from the same mice were also stained to demonstrate Gli2. Results from representative DMSO- and GDC-0449-treated mice are displayed. Quantitative Gli2 immunohistochemistry data were generated by counting nuclear Gli2 positive ductular and hepatocytic cells in tumor sections under 40× magnification. Results are graphed as mean±SEM Gli2-positive cells/40× high power field (**p<0.01) **C–D** Quantitative reverse transcription-PCR (qRT-PCR) analysis of whole liver RNA from DMSO-(open bar) and GDC-0449 (black bar) treated mice. **C.** PPAR-γ, a gene that is normally repressed by Hh signaling. **D.** Gli1, a gene that is induced by Hh signaling. Mean±SEM are graphed (**p<0.01).

Based on the data acquired in our dose-finding study, a second cohort of Mdr2^−/−^ mice (51–59 weeks of age) were assigned to treatment with vehicle (DMSO; n = 10) or 40 mg/kg GDC-0449 (n = 10) via daily i.p injection for 9 days. Overall survival between the two groups in the second cohort was equal, with no deaths in the DMSO treatment group and 1 death in the GDC-0449 treatment group secondary to iatrogenic injury. Both the liver parenchyma and tumors of treated mice showed decreased expression of the Hh pathway target Gli2 (**Figures 2A–B**). Real-time PCR analysis of resected tumors revealed that GDC-0449 treatment released the inhibitory effects of Hh signaling on PPARã, causing a significant increase in its gene expression (**Figure 2C**). Treatment with GDC-0449 also caused a significant decrease in expression of Gli1, another Hh target gene (**Figure 2D**).

### Hedgehog signaling inhibition with GDC-0449 reduces liver fibrosis

**Figure 3:**
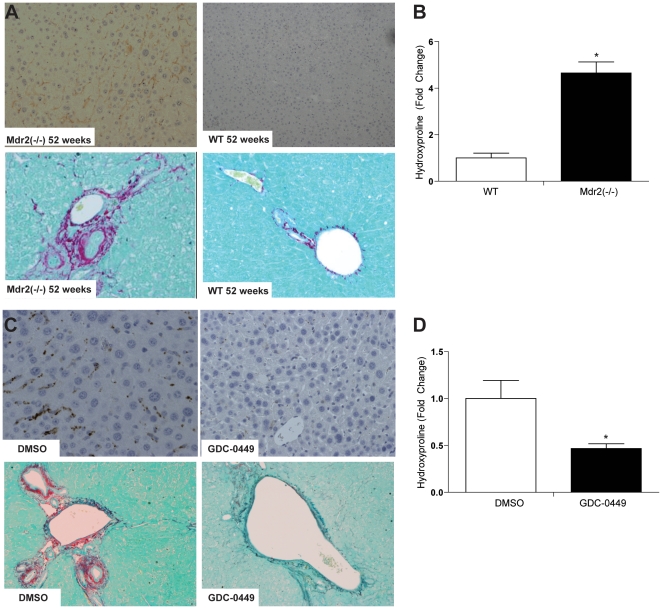
GDC-0449 treatment reduces fibrosis in Mdr2^−/−^ mice. **A.** Immunohistochemical staining for α-SMA (top panel) and Sirius red (bottom panel) in sections of non-tumor liver from representative age-matched Mdr2^−/−^ and wildtype mice (10×). **B.** Pooled Hepatic hydroxyproline content of 2–*52 wk-old* wildtype (WT) and age-matched Mdr2^−/−^ mice (n = 3–5/group). Results in Mdr2^−/−^ mice were normalized to that of age-matched WT mice and graphed as fold change. Data are displayed as mean +/− SD (*p<0.05) **C.** Non-tumor liver sections stained for α-SMA (top panel, 20×) and Sirius red (bottom panel, 10×) in representative DMSO- and GDC- treated Mdr2^−/−^ mice. **D.** Heptic hydroxyproline content of DMSO- and GDC- treated mice (n = 9/group). Results in GDC-0449-treated mice were normalized to that of DMSO vehicle-treated mice and graphed as fold change. Data are displayed as Mean +/− SEM (*p<0.05).

Mdr2^−/−^ mice demonstrated progressive age-related increases in hepatic expression of alpha-smooth muscle actin (α-sma), a marker of myofibroblastic hepatic stellate cells (**Figure 3A** **, top panel**). This was accompanied by enhanced expression of pro-fibrogenic factors, such as transforming growth factor TGF-β and platelet derived growth factor PDGF-β (**Figures S3A–B**), and progressive fibrosis, as evidenced by Sirius red staining (**Figure 3A** **, bottom panel**) and quantification of the hepatic hydroxyproline content (**Figure 3B**). Treatment with GDC-0449 decreased α-sma-expressing myofibroblastic cells, hepatic expression of TGF-β and PDGF-β, Sirius red staining, and hydroxyproline content, demonstrating that Hh pathway inhibition reduced liver fibrosis (**Figures 3C–D** **and Figures S3C–D**).

### Hedgehog signaling inhibition with GDC-0449 decreases accumulation of liver progenitor cells

**Figure 4:**
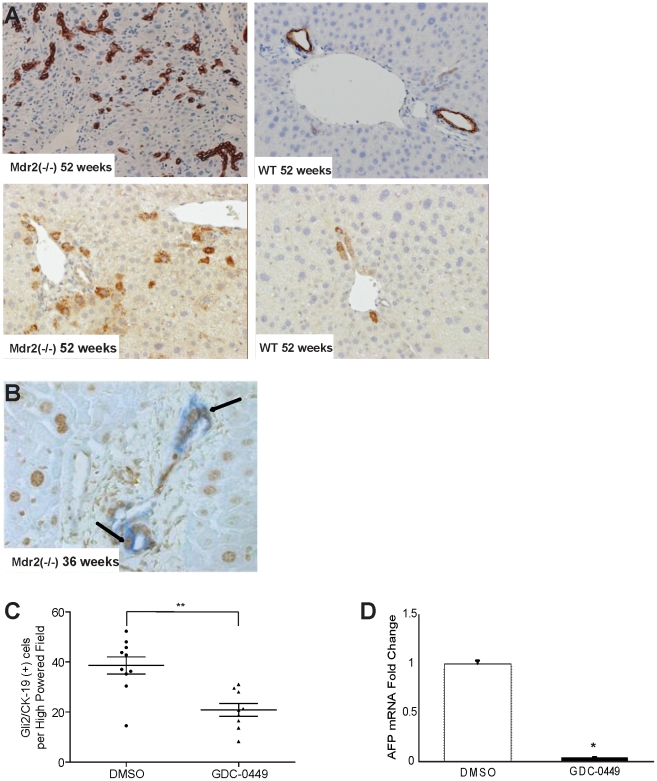
Effects of Mdr2 deficiency and Hedgehog (Hh) inhibition on hepatic progenitor populations. **A.** Immunohistochemical staining of liver sections from representative, age-matched Mdr2^−/−^ and wildtype mice for progenitor markers, cytokeratin-19 (CK-19) (top panels) and α -fetoprotein (AFP) (bottom panels) (20×). **B.** Representative micrograph of a portal triad in liver of an Mdr2^−/−^ mouse, demonstrating co-localization of CK-19 (blue) and Gli2 (brown) in the ductular compartment (40×). **C.** Quantitative Gli2 and CK19 immunohistochemistry in DMSO- and GDC-0449-treated Mdr2^−/−^ mice (n = 9–10/group). The number of Gli2 and CK19 double-positive ductular-appearing cells were counted within tumors under 20× magnification. Mean±SEM double(+) cells t per high power field (HPF) are graphed (* p<0.05) **D.** QRT-PCR analysis of AFP in tumor RNA from mice treated with DMSO (open bar) or GDC-0449 (closed bar) Results in the GDC-0449-treated mice were normalized to that of the mice treated with DMSO vehicle and graphed as Mean±SEM (*p<0.05).

In response to injury, progenitor populations in the liver proliferate. Hence, immunohistochemical analysis for progenitor markers, such as cytokeratin-19 (CK-19) and α-fetoprotein (AFP), showed age-related increases in Mdr2^−/−^ mice (**Figure 4A**). Co-staining for CK19 and Gli2 confirmed that the progenitor population was Hh responsive (**Figure 4B**). Upon treatment with GDC-0449, progenitor markers decreased (**Figure 4C**), indicating that Hh signaling was required to maintain progenitor populations during tumorigenesis, and that Hh inhibition was sufficient to shrink the size of progenitor populations even in mice with advanced HCC.

### Decreasing Hedgehog signaling reduces expression of osteopontin and prevents accumulation of osteopontin-responsive (CD44-positive) cells

**Figure 5:**
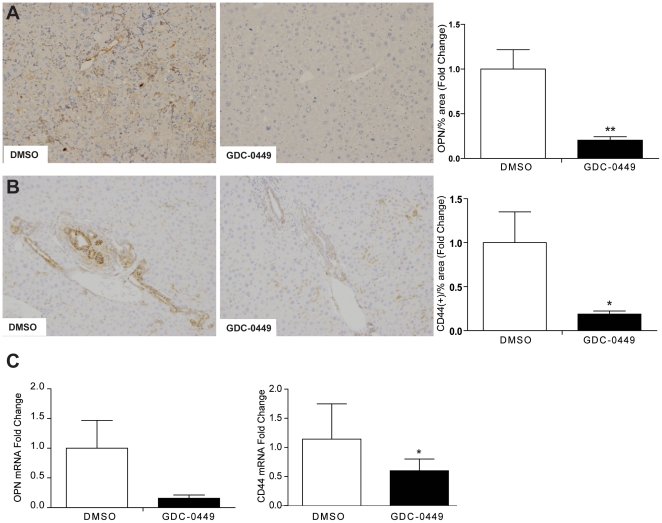
Inhibition of Hh signaling decreases osteopontin and osteopontin-responsive (CD44) positive cells in tumors and peri-tumoral tissues of aged Mdr2^−/−^ mice. **A.** Tumor sections from representative DMSO- and GDC-0449 treated Mdr2^−/−^ mice were stained to demonstrate osteopontin (OPN) Representative sections are displayed (*Right panel*). OPN staining was quantified by morphometric analysis of at least 5 HPF per tumor section using 20× magnification (n = 5 mice/group). Results in the GDC-0449-treated group were normalized to that of the group treated with DMSO vehicle and graphed as fold change. Data are displayed as Mean±SEM (**p<0.01). **B.** Immunohistochemical staining for the osteopontin receptor, CD44, in peri-tumoral tissues of representative DMSO- and GDC-0449- treated Mdr2^−/−^ mice. (*Right panel*) CD44 staining was quantified by morphometric analysis as described in A. Results in GDC-0449-treated mice were normalized to those of vehicle-treated controls and graphed as Mean±SEM (**p<0.01). **C.** QRT-PCR analysis of liver tumor RNA from DMSO- (open bar) and GDC-0449- (closed bar) treated Mdr2^−/−^ mice for OPN (left) and CD44 (right). After normalization to results in the DMSO-treated group, Mean±SEM were graphed (*p<0.05).

Stem/progenitor cell populations for many types of cancer, including HCC, are thought to be enriched with cells that express CD44, a receptor for the stem cell growth factor, osteopontin [35], [36]. Osteopontin expression is regulated by Hh signaling [37]. Therefore, we examined the effects of GDC-0449 on osteopontin and its receptor, CD44. Immunohistochemistry and quantitative morphometry demonstrated that inhibition of the Hh pathway with GDC-0449 significantly decreased osteopontin staining within primary liver tumors (**Figure 5A**). Similar treatment-related decreases in CD44+ tumor cells were also noted (**Figure 5B**). Gene expression analysis showed that expression of both osteopontin and CD44 mRNAs also tended to decrease in GDC-0449-treated mice (**Figure 5C**). Together, these results suggest that Hh signaling may regulate putative liver cancer stem/progenitor cells by modulating availability of osteopontin.

### MRI and histological evidence of liver tumor involution following GDC-0449 treatment

**Figure 6:**
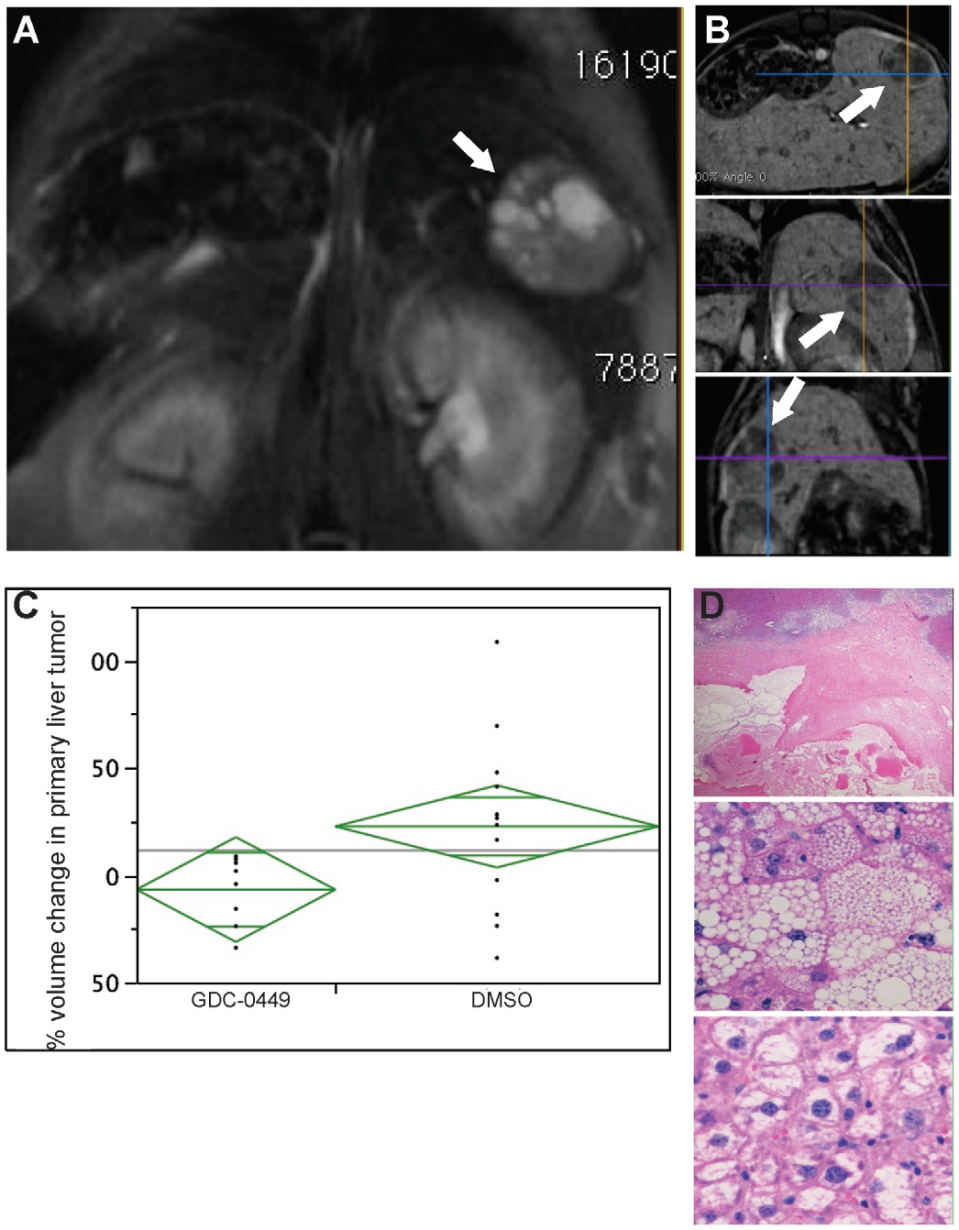
Hh pathway inhibition decreases in liver tumor volume by magnetic resonance imaging (MRI) and induces histologic features of tumor involution. **A.** Representative cross-sectional image of intrahepatic tumor as identified by contrast-enhanced MRI following GDC-0449 treatment. **B.** Representative MRI images of sagittal, coronal, and oblique perspectives used for volumetric analysis. **C.** Change in tumor volume were quantified by computerize generated volumetric measurements and graphed as Mean±SD (*p<0.05). **D.** Micrographs of representative histologic changes induced by, GDC-0449, treatment. Top panel shows tumor necrosis; middle panel demonstrates fatty degeneration; bottom panel show vacuolated tumor cells with pyknotic nuclei.

To evaluate the potential impact of changes in matrix and progenitor cells on HCC, pre- and post-treatment tumor volumes were analyzed using magnetic resonance imaging (**Figures 6A–B**). An analysis of tumors in mice without overt metastasis demonstrated that tumor volumes decreased in mice that received a 9 day course of GDC-0449-treatment, while vehicle-treated animals evidenced persistent tumor growth (**Figure 6C**; −6.7%+/−11.7% vs 22.7% +/−9.1%, p = 0.03). This data correlated with necropsy findings: Only 56% of GDC-0449-treated mice had visible liver tumor nodules, compared to 80% of the DMSO mice (**Table 1**). Furthermore, both MRI and necropsies showed a decreased number of metastasis in GDC-0449-treated mice compared to vehicle-treated controls (**Table 1**). Histological analysis of H&E-stained liver sections was also performed on all animals from both cohorts. If tumor nodules were not grossly visible or greater than 10 mm in size, the samples were excluded from analysis. Microscopic tumor nodules in GDC-0449 treated animals demonstrated increased rates of hemorrhagic infarct (20% vs 0%; **Figure 6D** **, top panel**), microvesicular steatosis (40% vs 0%, **Figure 6D** **, middle panel**), acidophilic necrosis and degenerative cytoplasmic changes (70% vs 40%, **Figure 6D** **, bottom panel**) in comparison to tumors from vehicle treated animals. Thus, findings on MRI, necropsy, and liver histology were consistent and demonstrated that Hh pathway inhibition caused significant regression of primary and metastatic HCC.

**Table 1:** Effect of GDC-0449 treatment on intrahepatic HCC and HCC metastases.

	Surgical Resection		MRI Imaging	
	Vehicle	Treatment	Vehicle	Treatment
**Intrahepatic Tumor Nodules**	80%	56%	78%	50%
**Metastases**	20%	0%	50%	20%

## Discussion

We studied Mdr2^−/−^ mice to determine whether or not activation of the Hh pathway contributes to hepatocarcinogenesis during chronic fibrosing liver injury. Mdr2-deficient mice lack a phospholipid flippase that is required for normal bile formation, and consequently exhibit liver injury and ductular proliferation from a young age. All afflicted mice eventually develop significant liver fibrosis and metastatic HCC. Our results demonstrate that this pathology is accompanied by progressive activation of the Hh pathway. Introduction of a specific inhibitor of the key Hh signaling intermediate, Smoothened, reduced pathway activity and proved that sustained Hh signaling was required to maintain the expanded populations of liver myofibroblasts and progenitors that had accumulated in the damaged livers. Moreover, treating aged Mdr2-deficient mice (which already had advanced liver fibrosis and HCC) with the Hh pathway inhibitor significantly reduced liver fibrosis and tumor burden, demonstrating for the first time that inhibiting Hh signaling has clinically-relevant, therapeutic value for both liver fibrosis and HCC. Even more exciting is evidence that the prohibitive effect on hepatic tumor growth *in vivo* pertains to both intra-hepatic HCC and distant metastasis.

In addition, our results provide insight into some of the underlying mechanisms involved. Hepatic production of Hh ligands was increased in mice that developed progressive liver fibrosis and invasive HCC. These mice also demonstrated consistently higher serum aminotransferase levels, in keeping with other evidence that Mdr2 deficiency provokes chronic hepatocyte injury [38], [39]. Various stressors that reduce hepatocyte viability have been shown to induce production and release of Hh ligands by wounded hepatocytes [40], [41]. Thus, hepatocyte injury is likely one of the factors that increases Hh ligand generation in mdr2-deficient livers. Myofibroblastic cells and ductular-type progenitors that progressively accumulate in chronically injured livers also produce Hh ligands, as well as other factors, such as PDGF-β, that stimulate autocrine-paracrine synthesis of Hh ligands [20], [42]. Relative to age/gender matched healthy control mice, aged mdr2-deficient mice with ductular reactions and liver fibrosis demonstrated higher hepatic mRNA levels of PDGF-β, suggesting a mechanism by which the fibrogenic repair response itself might perpetuate excessive hepatic accumulation of Hh ligands. The latter concept is supported by evidence that inhibiting Hh signaling with GDC-0449 reduced accumulation of myofibroblasts and ductular-type progenitors, decreased expression of PDGF-β, and suppressed hepatic expression Hh ligands in mice that remained at risk for hepatocyte injury due to genetic deficiency of Mdr2.

Chronic over-production of Hh ligands has important pathobiological implications because mice that generated increased Hh ligands also demonstrated excessive activation of the Hh pathway in their livers. This was evidenced by larger numbers of cells with nuclear staining for the Hh-regulated transcription factor, Gli2, and enhanced expression of various Hh-target genes, including Gli1 and osteopontin. In such animals, we showed that treatment with a highly specific antagonist of Smoothened was able to suppress all of these responses, consistent with published evidence that activation of Smoothened in Hh-responsive cells promotes nuclear localization of Gli2, and consequent induction of Gli1 and osteopontin transcription [43], [44]. Osteopontin, in turn, has been shown to promote myofibroblast accumulation and liver fibrosis in mice [37], [45]. It also acts via its receptor, CD44, to enhance the viability and growth of certain types of liver progenitors, including liver cancer stem/progenitor cells [35], [36], [46], [47]. Hence, the Hh pathway might be modulating both liver fibrosis and HCC outgrowth by regulating the autocrine/paracrine availability of osteopontin.

Myofibroblasts and ductular progenitor cells are also important sources of other pro-fibrogenic factors (e.g., PDGF-β and TGF-β) that are capable of activating Gli2 via mechanisms that do not require Smoothened [48], [49], [50]. We observed increased hepatic expression of both PDGF-β and TGF-β in Mdr2-deficient mice. This suggests that Smoothened-independent mechanisms that re-enforce the effects of the canonical Hh pathway may evolve during fibrogenic repair. In mdr2-deficient mice, however, GDC-0449 significantly reduced expression of TGF-β and tended to suppress expression of PDGFβ, demonstrating that canonical Hh signaling with resultant Smoothened activation is ultimately required to fully engage noncanonical pathways that rely on interaction of TGF-β and PDGF-β with their respective receptors to activate Gli2 in injured, fibrotic livers.

Finally, the fact that liver fibrosis/cirrhosis is a major risk factor for the development of HCC should not be misconstrued to imply that fibrosis *per se* causes cancer. It is conceivable that fibrosis and HCC each result from some adaptive response that occurs during chronic liver injury. Our results demonstrate that injury-related activation of the Hh pathway typifies an adaptive response that is both pro-fibrogenic and pro-carcinogenic. This discovery, in turn, provides novel evidence that helps to explain why fibrosis and HCC often develop in the same livers. As such, it has immediate clinical relevance to the many cirrhotic patients with HCC. Additional research will be required, however to delineate the precise down-stream mechanism(s) involved and to determine which (if any) of those subsequent fibrogenic and carcinogenic processes are inter-dependent, and which are totally independent of each other.

Proof that inhibition of Hh signaling substantially reduced the hepatic content of myofibroblasts and progenitors suggests that these cell types promote and/or maintain the outgrowth of malignant hepatocytes. This finding, in turn, provides a starting point for further research. Indeed, others have reported that Smoothened antagonists lead to the involution of pancreatic cancers by influencing tumor angiogenesis [51]. Myofibroblasts are known to be an important source of vascular growth factors, such as VEGF [52], [53], [54], and we found increased hepatic expression of VEGF and its receptor, VEGFR1, in the diseased livers of Mdr2-deficient mice compared to the healthy livers of age/gender-matched controls (data not shown). Hence, it is possible that myofibroblast depletion exerted a negative impact on the hepatic vasculature that ultimately resulted in HCC involution. This concept is supported by evidence that GDC-0449 tended to suppress hepatic expression of VEGF/VEGFR1 (data not shown) and increased necrosis in the HCC of GDC-0449-treated mice. Hh signaling is also known to maintain various progenitor populations [55], [56], [57]. Previously, we reported that Hh ligands function as autocrine and paracrine survival signals for liver progenitors and showed that liver myofibroblasts are an important paracrine source of Hh ligands that serve this purpose [40], [42], [58], [59], [60]. Liver progenitors, in turn, generate Hh ligands that provide paracrine signals that re-enforce the growth of liver myofibroblasts [61], [62]. Given this background, it is not surprising that blocking Hh signaling with a Smoothened antagonist resulted in the mutual depletion of both cell types. To our knowledge, however, by demonstrating marked treatment-related decreases in CD44, cytokeratin-19, and α-fetoprotein within HCC tumors, the current data provide the first evidence that Smoothened antagonists reduce populations of cells that exhibit features of tumor stem/progenitor cells. Further investigation is required to ascertain how aberrant Hedgehog signaling promotes these cancer stem cell compartments.

In conclusion, our findings in the Mdr2^−/−^ mouse model of progressive liver fibrosis and spontaneous hepatocarcinogenesis demonstrate that increased production of Hh ligands and progressive accumulation of Hh-responsive cell types, such as myofibroblasts and liver progenitor cells, precede the emergence of HCC, and persist after the development of HCC. Despite advanced liver fibrosis and HCC, a short course of treatment with a highly-specific and clinically available Hh signaling inhibitor is well-tolerated and demonstrates appreciable anti-tumor effects. Thus, Hh pathway inhibition with GDC-0449 merits evaluation as a potential treatment for HCC arising in cirrhotic patients, providing a novel treatment option for an emerging disease with a poor prognosis and limited therapeutics.

## Supporting Information

Figure S1 **Evidence of ongoing liver injury in Mdr2^−/−^ mice.** AST and ALT measurements from Mdr2^−/−^ mice and their age-matched wild type counterparts at various time points. Each data point represents n = 2–7 animals and Mean±SD is graphed. (*p<0.05 vs the age-matched wild-type control).
(TIFF)

Figure S2 **Systemic treatment of GDC-0449 treatment is well tolerated in Mdr2^−/−^ mice with advanced liver disease and HCC.** **A.** Daily body weight measurements were obtained for all animals in each of the three treatment groups and graphed over time. Data expressed as Mean±SD. **B.** Liver to body weight ratios of animals in each of the three treatment groups. Data expressed as Mean±SD. **C.** Western blot analysis for Shh, Gli1, Gli2 and actin (loading control) in whole liver extracts from Mdr2−/− mice treated with vehicle, 20 mg/kg GDC-0449, and 40 mg/kg GDC-0449. Liver extracts from each treated mouse were loaded individually.
(TIFF)

Figure S3 **Increased hepatic expression of TGFβ and PDGFβ in Mdr2^−/−^ mice is reversed by GDC-0449 treatment.** Whole liver RNA was isolated from Mdr2^−/−^ mice and age/gender-matched wild type controls (WT) (n = 3 mice/group) and QRT PCR was done to compare expression of (**A**) TGFβ and (**B**) PDGFβ. Similar approaches were used to assess the effects of a 9 day course of treatment with either the Hh pathway inhibitor GDC-0449 or vehicle (DMSO) on expression of (**C**) TGFβ and (**D**) PDGFβ in Mdr2^−/−^ mice (n = 5 mice/group). Gene expression was normalized to expression of S9 in the same samples; mean +/− SEM values were calculated; values in the experimental groups were graphed relative those in the respective controls. P values are shown.
(TIF)

Table S1 (PDF)
