# Supplementary figures and images for: Hedgehog Signaling Antagonist Promotes Regression of Both Liver Fibrosis and Hepatocellular Carcinoma in a Murine Model of Primary Liver Cancer
Source: PLoS One. 2011 Sep 2;6(9):e23943. doi: 10.1371/journal.pone.0023943 (PMC3166282; doi:10.1371/journal.pone.0023943)

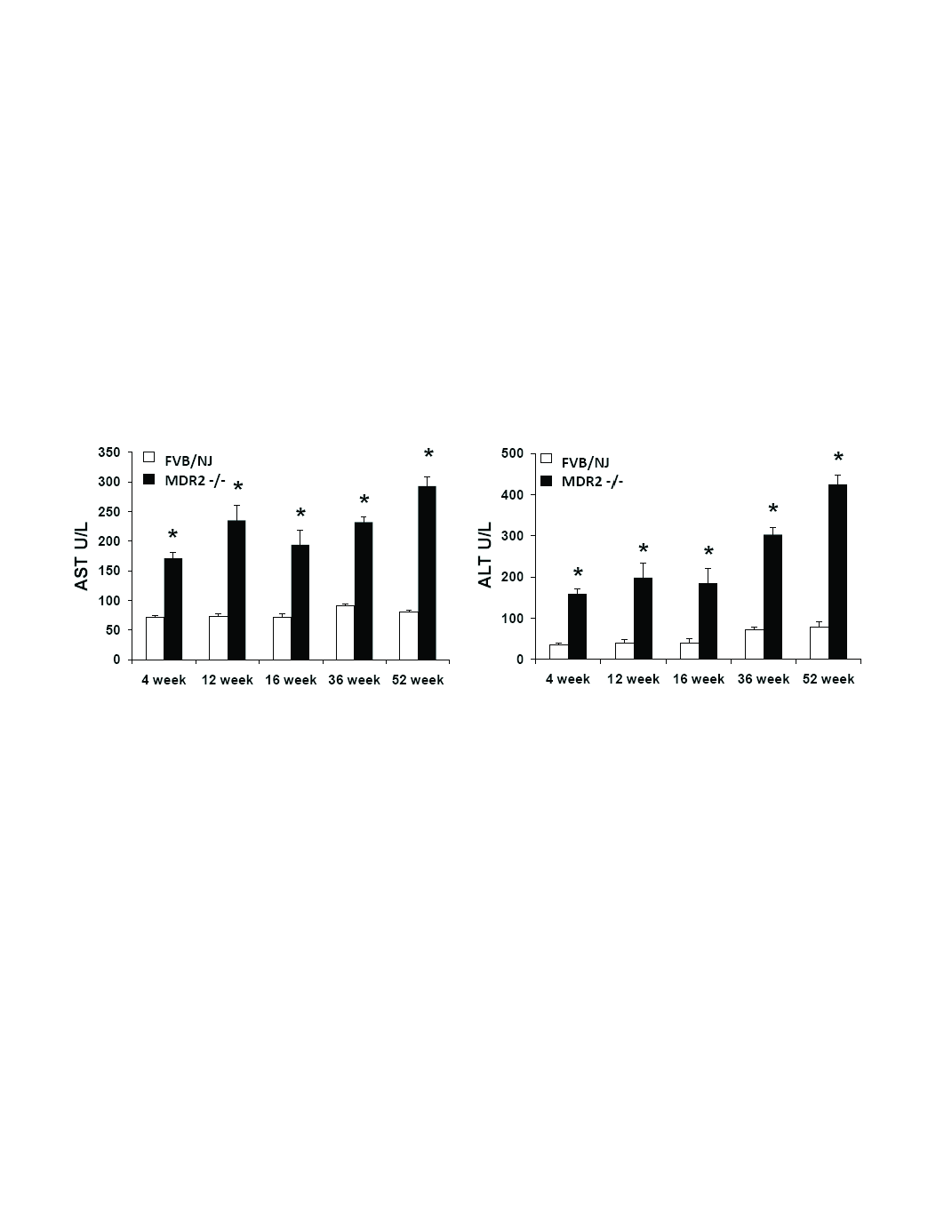

Supplement: Figure S1 — Evidence of ongoing liver injury in Mdr2−/− mice. AST and ALT measurements from Mdr2−/− mice and their age-matched wild type counterparts at various time points. Each data point represents n = 2–7 animals and Mean±SD is graphed. (*p<0.05 vs the age-matched wild-type control). (TIFF) [file pone.0023943.s001.tiff]

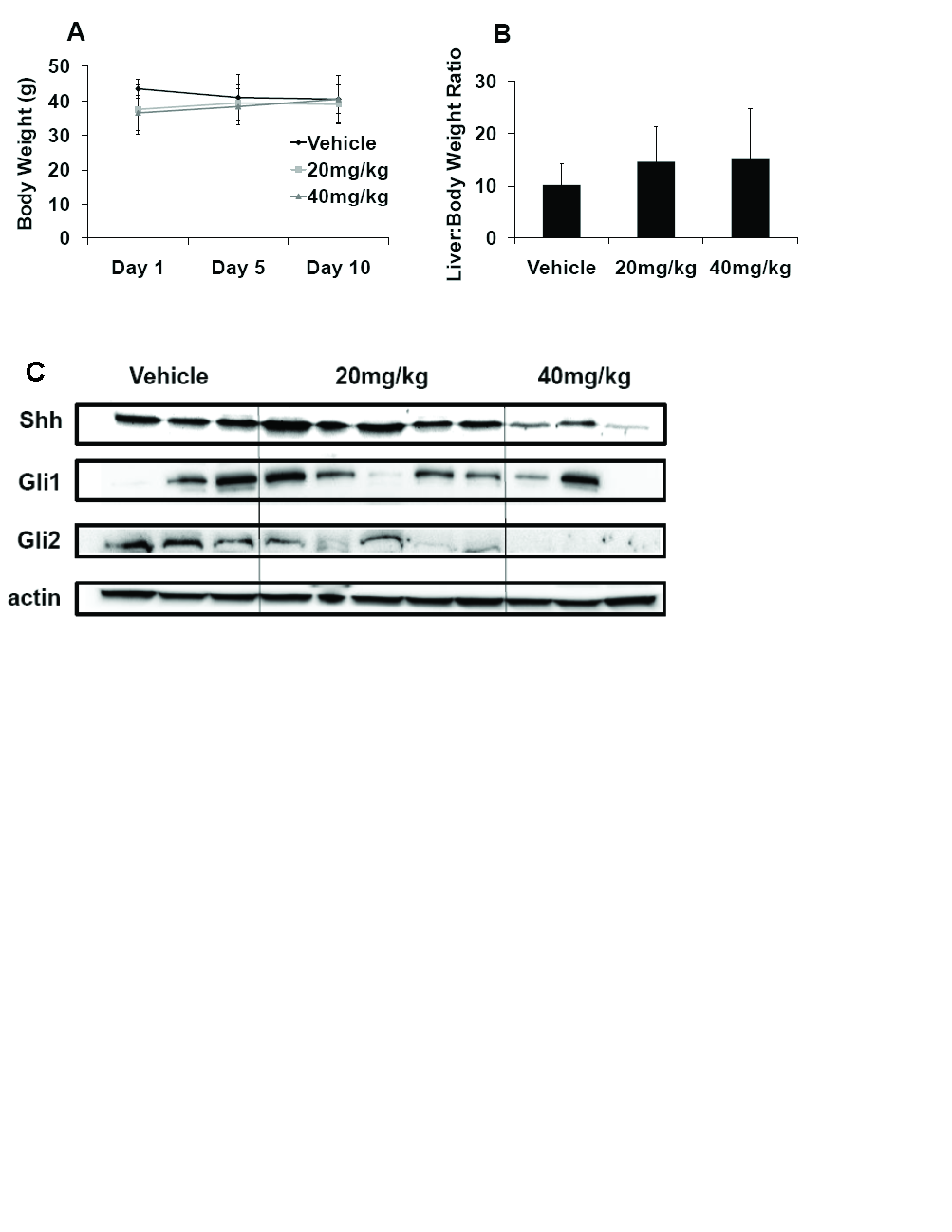

Supplement: Figure S2 — Systemic treatment of GDC-0449 treatment is well tolerated in Mdr2−/− mice with advanced liver disease and HCC. A. Daily body weight measurements were obtained for all animals in each of the three treatment groups and graphed over time. Data expressed as Mean±SD. B. Liver to body weight ratios of animals in each of the three treatment groups. Data expressed as Mean±SD. C. Western blot analysis for Shh, Gli1, Gli2 and actin (loading control) in whole liver extracts from Mdr2−/− mice treated with vehicle, 20 mg/kg GDC-0449, and 40 mg/kg GDC-0449. Liver extracts from each treated mouse were loaded individually. (TIFF) [file pone.0023943.s002.tiff]

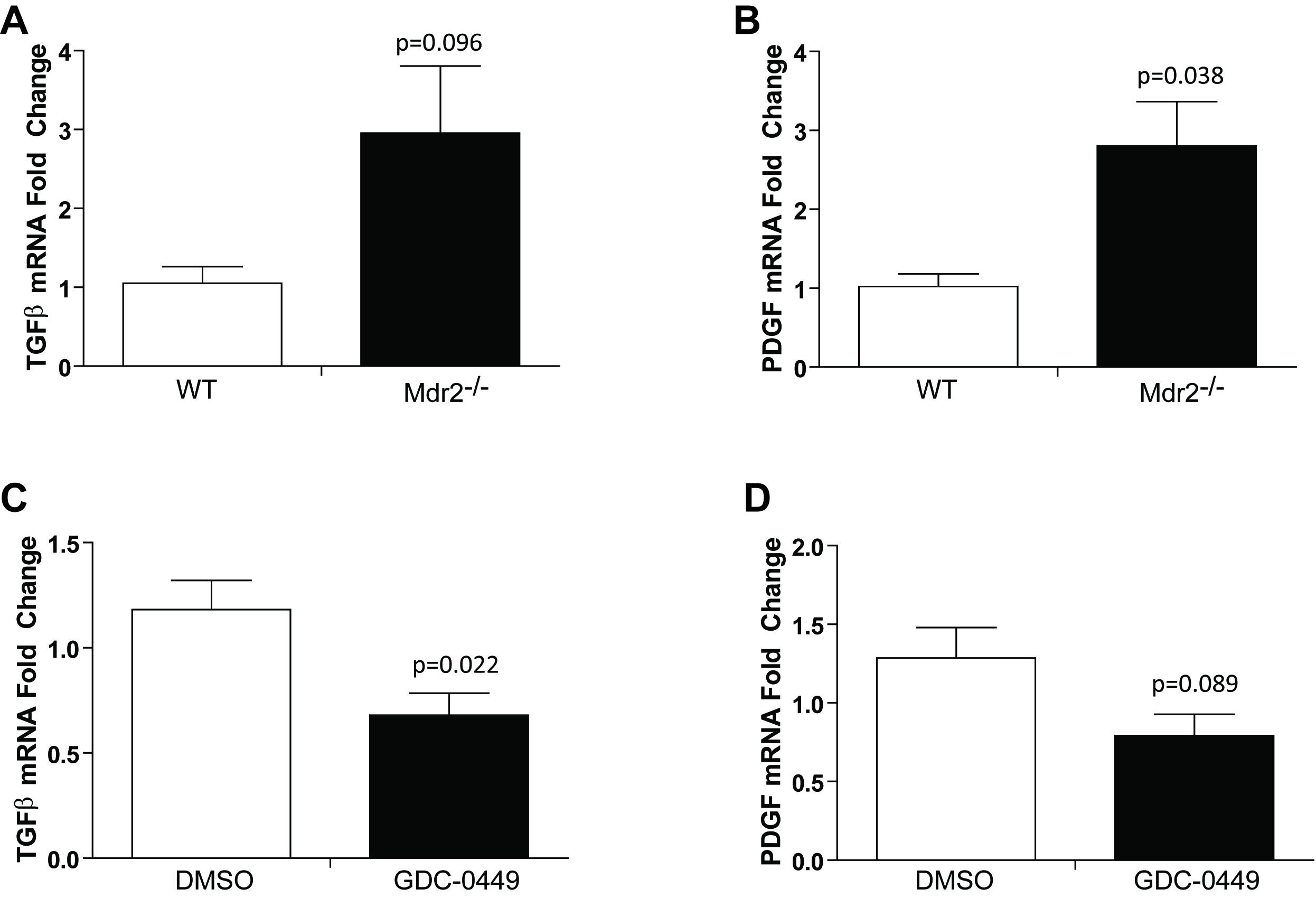

Supplement: Figure S3 — Increased hepatic expression of TGFβ and PDGFβ in Mdr2−/− mice is reversed by GDC-0449 treatment. Whole liver RNA was isolated from Mdr2−/− mice and age/gender-matched wild type controls (WT) (n = 3 mice/group) and QRT PCR was done to compare expression of (A) TGFβ and (B) PDGFβ. Similar approaches were used to assess the effects of a 9 day course of treatment with either the Hh pathway inhibitor GDC-0449 or vehicle (DMSO) on expression of (C) TGFβ and (D) PDGFβ in Mdr2−/− mice (n = 5 mice/group). Gene expression was normalized to expression of S9 in the same samples; mean +/− SEM values were calculated; values in the experimental groups were graphed relative those in the respective controls. P values are shown. (TIF) [file pone.0023943.s003.tif]
