# Supplementary material for: Hedgehog Signaling Antagonist Promotes Regression of Both Liver Fibrosis and Hepatocellular Carcinoma in a Murine Model of Primary Liver Cancer
Source: PLoS One. 2011 Sep 2;6(9):e23943. doi: 10.1371/journal.pone.0023943 (PMC3166282; doi:10.1371/journal.pone.0023943)
Supplement: Table S1 — (PDF) [file pone.0023943.s004.pdf]

Supplementary Table 1

Mouse primers:

S9	F	GACTCCGGAACAAACGTGAGGT
	R	CTTCATCTTGCCCTCGTCCA
AFP	F	GGC TTT CTA AAC ACC CAT CG
	R	CCG AGG AGG AAG TGA AAC AAA
PPAR-γ	F	ACT GGC ACC CTT GAA AAA TG
	R	CCC TGG CAA AGC ATT TGT AT
Gli2	F	ACCATGCCTACCCAACTCAG
	R	CTGCTCCTGTGTCAGTCCAA
OPN	F	CTC CAT CGT CAT CAT CAT CG
	R	TGC ACC CAG ATC CTA TAG CC
CD44	F	AGC GGC AGG TTA CAT TCA AA
	R	CAA GTT TGG GTG GCA CAC AG
TGFβ	F	TGG AGC AAC ATG TGG AAC TC
	R	CGT CAA AAG ACA GCC ACT CA
PDGFβ	F	GAC TAC CTG CAC CGG AAC AA
	R	GTG CAA CAT GGG CAC GTA A
